# Machine learning and deep learning to identifying subarachnoid haemorrhage macrophage‐associated biomarkers by bulk and single‐cell sequencing

**DOI:** 10.1111/jcmm.18296

**Published:** 2024-05-04

**Authors:** Sha Yang, Yunjia Hu, Xiang Wang, Mei Deng, Jun Ma, Yin Hao, Zhongying Ran, Tao Luo, Guoqiang Han, Xin Xiang, Jian Liu, Hui Shi, Ying Tan

**Affiliations:** ^1^ Department of Neurosurgery The Affiliated Hospital of Guizhou Medical University Guiyang China; ^2^ Guizhou University Medical College Guiyang China; ^3^ Department of Neurosurgery Guizhou Provincial People's Hospital Guiyang China; ^4^ Department of Neurosurgery Yongchuan Hospital affiliated to Chongqing Medical University Chongqing China

**Keywords:** deep learning, hdWGCNA, machine learning, single‐cell sequencing, subarachnoid haemorrhage rat model

## Abstract

We investigated subarachnoid haemorrhage (SAH) macrophage subpopulations and identified relevant key genes for improving diagnostic and therapeutic strategies. SAH rat models were established, and brain tissue samples underwent single‐cell transcriptome sequencing and bulk RNA‐seq. Using single‐cell data, distinct macrophage subpopulations, including a unique SAH subset, were identified. The hdWGCNA method revealed 160 key macrophage‐related genes. Univariate analysis and lasso regression selected 10 genes for constructing a diagnostic model. Machine learning algorithms facilitated model development. Cellular infiltration was assessed using the MCPcounter algorithm, and a heatmap integrated cell abundance and gene expression. A 3 × 3 convolutional neural network created an additional diagnostic model, while molecular docking identified potential drugs. The diagnostic model based on the 10 selected genes achieved excellent performance, with an AUC of 1 in both training and validation datasets. The heatmap, combining cell abundance and gene expression, provided insights into SAH cellular composition. The convolutional neural network model exhibited a sensitivity and specificity of 1 in both datasets. Additionally, CD14, GPNMB, SPP1 and PRDX5 were specifically expressed in SAH‐associated macrophages, highlighting its potential as a therapeutic target. Network pharmacology analysis identified some targeting drugs for SAH treatment. Our study characterised SAH macrophage subpopulations and identified key associated genes. We developed a robust diagnostic model and recognised CD14, GPNMB, SPP1 and PRDX5 as potential therapeutic targets. Further experiments and clinical investigations are needed to validate these findings and explore the clinical implications of targets in SAH treatment.

## 1 INTRODUCTION

Subarachnoid haemorrhage (SAH) is characterised by bleeding in the subarachnoid space, commonly caused by intracranial aneurysm rupture.^1^ SAH has a significant impact on public health, with an estimated annual incidence of 6–16 cases per 100,000 people. Mortality rates are high, with 30% of patients dying within 24 h and an additional 40% experiencing long‐term disabilities.^2^ SAH management remains challenging, and while diagnostic techniques and treatment strategies have improved, there is room for advancement. Current treatments include endovascular coiling or surgical clipping to secure the ruptured aneurysm, but limitations exist, and the choice depends on aneurysm characteristics and patient‐specific factors.^3^

Macrophages play a significant role in SAH, involved in mechanisms such as inflammation, blood component clearance and tissue repair.^4^ Studies have shown their regulatory role in SAH‐associated inflammation, with activated macrophages releasing inflammatory mediators and contributing to brain injury.^5^ Immunohistochemistry and molecular biology techniques have provided a detailed understanding of macrophage inflammatory response. Macrophages also play a crucial role in clearing blood components, demonstrated by dense aggregation in the subarachnoid space and participate in tissue repair and regeneration processes through growth factor secretion.^6^ Transcriptomic analysis and immunohistochemical techniques have identified signalling pathways and molecular mechanisms.^7^ Further exploration of macrophage mechanisms in SAH treatment and intervention is vital.

Single‐cell transcriptomic sequencing, bioinformatics analysis, and machine learning/deep learning algorithms are essential for studying macrophages in SAH.^8^, ^9^ These techniques offer detailed single‐cell gene expression information, enhancing our understanding of their critical role in SAH. Single‐cell transcriptomic sequencing reveals macrophage subpopulations, functional differences and transcriptional profiles.^8^, ^9^, ^10^ Analysis of single‐cell data identifies macrophage‐specific gene expression patterns associated with inflammation, apoptosis and repair, exploring relevant signalling pathways.^8^, ^9^, ^10^ Bioinformatics analysis deciphers macrophage interactions, integrating data with biological databases to identify signalling pathways and regulatory networks. This sheds light on macrophage function in SAH development and the inflammatory response. Machine learning and deep learning algorithms extract valuable patterns and information from large‐scale data, identifying potential SAH‐related biomarkers for personalised treatment and precision medicine.^11^, ^12^

In this study, we utilised a rat model of SAH, performing single‐cell transcriptomic sequencing and bulk RNA‐seq on collected brain tissue. Integrating public gene expression databases, we identified macrophage features in SAH using single‐cell transcriptomic data. Machine learning and deep learning algorithms were employed to build a diagnostic model based on bulk RNA‐seq data, identifying characteristic gene expression patterns in SAH patients. Additionally, network pharmacology methods explored potential therapeutic drugs targeting macrophages. Through these research methods and analytical strategies, our aim is to deepen our understanding of macrophage involvement in SAH mechanisms, providing new perspectives and strategies for SAH diagnosis and treatment.

## 2 MATERIALS AND METHODS

### 2.1 Animal

All experimental procedures were approved by the Institutional Animal Care and Use Committee (IACUC) of Guizhou Provincial People's Hospital (No.). A group of healthy adult male Sprague–Dawley rats (*n* = 12; age = 8 weeks; weight = 280–300 g) were housed in a temperature‐ and light‐controlled room with ad libitum access to food and water. The rats were fed standard laboratory chow with an average daily intake of approximately 20 g per rat and provided with sterile water that was changed regularly. The animals were maintained under a 12‐h light–dark cycle at a temperature of 22 ± 2°C and a humidity of 50 ± 5%. Prior to modelling, the rats were randomised into Sham and SAH groups in a 1:1 ratio.

### 2.2 SAH model and study design

Subarachnoid haemorrhage was induced in rats through endovascular puncture.^13^ Briefly, the rats were initially anaesthetized with 5% isoflurane for induction and subsequently maintained under 2%–3% isoflurane after intubation. The right common carotid artery, external carotid artery, and internal carotid artery were meticulously exposed and separated. The external carotid artery was then ligated and severed. A 4–0 surgical nylon suture was carefully inserted from the external carotid artery into the internal carotid artery until resistance was encountered. Subsequently, the suture was advanced by 2–3 mm to perforate the vessel.

The identical surgical procedure was conducted in the sham group, with the exception of omitting the vascular puncture. Following the surgery, the rats were housed in individual warming cages until they regained consciousness.

**FIGURE 1:**
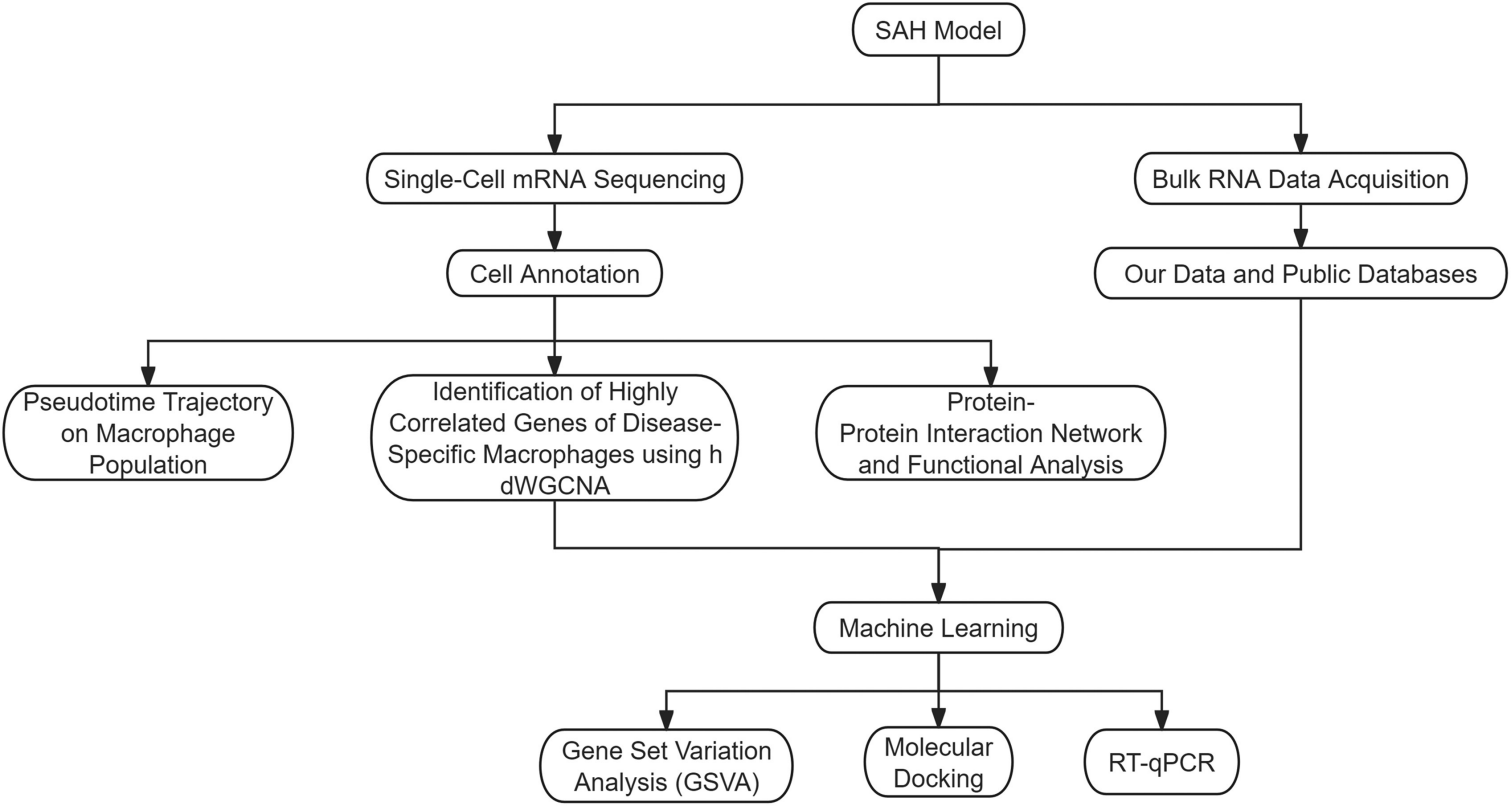
The flow chart of the study.

Figure 1 clearly delineated the research process.

### 2.3 Tissue dissociation and cell isolation

Twenty‐four hours post‐surgery, rats were anaesthetized, and saline perfusion was conducted to isolate the tissue. SAH grading on the brain was carried out by two independent researchers using the Sugawara grading system,^14^ where the basal cistern was divided into six regions scored from 0 to 3. Subsequently, the right temporal cortex was harvested and transferred to a sterile RNase‐free culture dish containing calcium‐free and magnesium‐free 1 × PBS on ice.

The tissue was then cut into 0.5 mm^2^ pieces, washed with 1 × PBS, and non‐essential tissues, such as blood stains and fatty layers, were carefully removed. Tissue pieces underwent dissociation into single cells utilising a dissociation solution comprised of 0.35% collagenase IV, 5.2 mg/mL papain and 120 Units/mL DNase I. This was followed by incubation in a 37°C water bath with shaking for 20 minutes at 100 rpm. Digestion was terminated with 1 × PBS containing 10% foetal bovine serum (FBS, V/V), and the resulting cell suspension was filtered and centrifuged.

The cell pellet was then resuspended in 1 × PBS (0.04% BSA), and dead cells were eliminated using a Dead Cell Removal Kit. Overall cell viability was confirmed by trypan blue exclusion, and single‐cell suspensions were enumerated using a Countess II Automated Cell Counter. The concentration was subsequently adjusted to a range of 700–1200 cells/μL.

### 2.4 Single‐Cell mRNA Sequencing

Single‐cell suspensions were loaded onto the 10x Chromium system (10x Genomics) following the manufacturer's guidelines for the Chromium Single‐Cell 3′ kit (V3) to capture 5000 single cells. The captured cells underwent cDNA amplification and library construction steps in accordance with the standard protocol outlined in the Chromium Single‐Cell 3′ kit (V3) from 10x Genomics.

In brief, the captured cells were lysed, and the released RNA was reverse‐transcribed into cDNA. Subsequently, the cDNA underwent amplification, fragmentation and ligation with adapters to prepare the sequencing library. To ensure the libraries met the minimum depth requirement of 20,000 reads per cell for downstream analysis, quality control measures were implemented. The libraries were sequenced on an Illumina NovaSeq 6000 sequencing system (paired‐end multiplexing run, 150 bp) by LC‐Bio Technology Co. Ltd. (Hangzhou, China).

The resultant reads underwent quality control using the 10x Cell Ranger software package (v1.2.0; 10x Genomics), aligning them to the mm10 reference genome assembly (v1.2.0; 10x Genomics). A total of 4000 cell barcodes were reported, with a median of 1105 genes per cell, sequencing saturation of 93.4%, and an average read count of 91,009 per cell. For the HDM/LPS + vehicle‐treated sample, 4034 cell barcodes were reported, with a median of 1018 genes per cell, sequencing saturation of 93.5% and an average read count of 129,637 per cell.

### 2.5 Analysis of single‐cell RNA sequencing

The Cell Ranger software package (version 2.1) from 10x Genomics was utilized for demultiplexing, alignment to the human reference genome GRCh38 and unique molecular identifier (UMI) collapsing. Quality control filtering was implemented to exclude cells with fewer than 500 reads, fewer than 200 genes, or a proportion of mitochondrial reads exceeding 25%. Doublets in the data were predicted using DoubletFinder and subsequently filtered out.

The “LogNormalize” method in the Seurat^15^ R software was then employed for the normalisation of each individual sample. The “FindVariableFeatures” function was applied to select a set of 2500 highly variable genes. To overcome batch effects across multiple samples, the “harmony” method was utilised, and the first 20 principal components were chosen for integration. The “FindClusters” function identified clusters by calculating a shared neighbour graph with a resolution of 0.6. Cell‐type annotation was performed using the SingleR^16^ package with reference markers.

Clustering results were visualized using the uniform manifold approximation and projection (UMAP) technique. Proportions of various cell types in the SAH and sham groups were compared, and macrophage populations were selected for subsequent analysis.

Monocytes were extracted, and the aforementioned dimensionality reduction, clustering, and batch removal algorithms were repeated. The first 15 principal components were selected for integration, and the “FindClusters” function was employed to differentiate macrophage subclusters. Specific macrophage subtypes, distinguishing the SAH group from the sham group, were identified as SAH‐specific macrophages (SSM).

To understand interactions among cells involving ligands, receptors and cofactors, analysis was conducted using the CellChat R package.^17^

### 2.6 Identification of highly correlated genes of disease‐specific cell subclusters using hdWGCNA

To pinpoint highly correlated genes within disease‐specific cell subclusters, we employed the hierarchical deconvolution‐based Weighted Gene Co‐expression Network Analysis (hdWGCNA)^18^ computational approach on single‐cell RNA sequencing (scRNA‐seq) data. Initially, disease‐specific cell subclusters were identified using a graph‐based clustering algorithm based on shared nearest neighbours. Subsequently, hdWGCNA was applied to the expression data of these cell subclusters to unveil highly correlated gene modules.

In detail, we constructed a signed co‐expression network using a soft threshold power and delineated gene modules by grouping highly correlated genes and employing a topological overlap measure. Module‐trait relationships were then leveraged to identify modules highly correlated with the disease‐specific cell subclusters. The top genes within these modules were subsequently selected as potential candidates for further functional studies. This approach enabled us to identify a set of genes intricately linked to the disease‐specific cell subclusters, thereby providing a valuable resource for future investigations aimed at unravelling the molecular mechanisms underlying the disease.

### 2.7 Pseudotime trajectory on macrophage population

Pseudotime analysis was carried out using the monocle R package,^19^ utilizing default settings, specifically focused on the macrophage population. The reduce dimension function was employed for pseudotime ordering, with max_components set to two and the reduction method set to DDRTree. Subsequently, we identified key genes related to SSM from the hdWGCNA analysis and depicted their expression profiles along the pseudotime trajectory using the plot pseudotime heatmap function. The resultant pseudotime trajectory plot facilitated the visualisation of gene expression changes in the macrophages over time.

### 2.8 Protein–protein interaction network and functional analysis

Key genes associated with SSM were identified through hdWGCNA analysis. To elucidate the interactions among these genes, a protein–protein interaction (PPI) network was constructed using the STRING database, employing a cut‐off of 0.9 to ensure high confidence in the interactions. Additionally, for a more comprehensive understanding of the roles played by these key genes in biological processes, Gene Ontology (GO) and Kyoto Encyclopedia of Genes and Genomes (KEGG) pathway enrichment analyses were conducted.

In the GO analysis, scrutiny was given to the involvement of these genes in molecular functions, biological processes and cellular components. Regarding the KEGG analysis, focus was directed toward the pathways in which these genes participate. Both the GO and KEGG analyses were executed using the clusterProfiler R package,^20^ with a statistical enrichment threshold set at *p* < 0.05.

### 2.9 Bulk RNA data acquisition

Following the outlined methodology, we acquired three samples of the right temporal cortex from rats in both the SAH and sham groups. Initially, brain tissues underwent washing in PBS buffer to eliminate blood and other contaminants. Subsequently, the brain tissues were finely sectioned and centrifuged in a tube for separation. Total RNA extraction from the samples was performed using Trizol reagent, and the quantity and purity of the total RNA were assessed using a Bioanalyzer 2100 with an RNA 6000 Nano Chip kit. High‐quality RNA, possessing a RIN value greater than 7.0, was selectively chosen for library construction.

For sequencing, mRNA was extracted from the total RNA using Oligo (dT) and subsequently fragmented into small pieces under conditions of high temperature and Mg2+. These fragmented RNA pieces were reverse‐transcribed into complementary cDNA using reverse transcriptase. Following this, a second DNA strand with a U label was synthesized in a dUTP solution using E. coli DNA polymerase and RNAase. To connect the DNA fragments and adapters, an adapter with a T base was added, and an A was added after the end of DNA synthesis to link the adapter to the A‐tailed DNA. Subsequently, magnetic beads were employed for size selection, connecting the double‐stranded adapters to the DNA fragments. The connected products were then amplified using PCR, which included initial denaturation, 8 cycles of denaturation, annealing and extension and a final extension. The resulting cDNA library had a size of 300 ± 50 bp, and paired‐end sequencing was conducted using the Illumina Novaseq platform.

Our transcriptome matrix served as a validation set for the diagnostic model. Additionally, to enhance the robustness of our model, we performed a systematic search in the GEO database (https://www.ncbi.nlm.nih.gov/geo/) to identify SAH expression datasets. The GSE36791 dataset from the GEO database was employed as the training set for our diagnostic model. This dataset comprises 43 SAH samples and 18 control samples, prospectively recruited from patients admitted to the Departments of Neurology or Neurosurgery and Neurotraumatology, University Hospital, Krakow, Poland in 2010 and 2011.

### 2.10 Machine learning

Building upon the key genes associated with SSM identified through hdWGCNA analysis, we conducted a screening process to identify key feature genes for SAH. To effectively reduce dimensionality and select the most predictive genes, we employed univariate logistic regression and LASSO regression methods.

Subsequently, we constructed a diagnostic model utilising seven distinct machine learning algorithms, namely logistic regression, linear discriminant analysis, support vector machine, naive Bayes, k‐nearest neighbours, decision tree and random forest. During the model training phase, the GSE36791 dataset was chosen as the training set, and the best‐performing model was selected by comparing the performance of various models.

Finally, model validation was carried out using our proprietary transcriptome sequencing data, thereby ensuring the robustness and applicability of the developed machine learning‐based diagnostic model.

### 2.11 Gene set variation analysis (GSVA)

The key genes identified from hdWGCNA analysis were utilized as the background gene set, and the scores of this background gene set in the SAH and control groups of the GSE36791 dataset were assessed using GSVA.^21^ The resultant score, referred to as the SAH macrophage score, was defined, and the disparity in scores between the two groups was examined.

Using the “IOBR::deconvo_mcpcounter” function,^22^ immune cells in both the self‐test transcriptome data and GSE36791 dataset samples were evaluated. A heatmap illustrating the correlation between genes and immune cells across the seven machine learning models was generated. Furthermore, a diagnostic atlas was created for each sample based on the genes and immune cells within the model. Subsequently, a 3 × 3 convolutional neural network was employed to construct a diagnostic model utilizing the information from the diagnostic atlas.

### 2.12 Molecular docking

Several genes were selected from those identified as key genes related to SSM in hdWGCNA analysis, specifically genes significantly expressed in macrophages of the SAH group but not in the sham group. UMAP plots were utilized to visually represent the expression patterns of these genes in macrophages.

To investigate the interaction information between these genes and chemicals, along with the list of diseases related to these genes, the CTDbase database was consulted. Molecular docking, a method for analysing the interaction between small‐molecule ligands and large protein targets, was then employed in this study.

Initially, the 2D structure of small‐molecule ligands was obtained from the PubChem database and converted into 3D structures using Chem3D software. The resulting structures were exported in mol2 format and further processed with AutoDockTools‐1.5.6 software to obtain pdbqt format. The large protein targets were sourced from the RCSB Protein Data Bank (PDB) and underwent preprocessing with PyMOLWin software to remove solvents and organic molecules.

The processed small‐molecule ligands and large protein targets were subsequently imported into Vina software for molecular docking, and the affinity between them was calculated to evaluate their interaction capability. Typically, an affinity value less than −5 kcal/mol indicates a strong interaction capability.

### 2.13 RT‐qPCR

TRIzol reagent (ThermoFisher Scientific Corporation, Shanghai, China) was used to extract total RNA from the right temporal lobe brain tissue of rats 24 h after SAH, and the extracted RNA (1 μg) was reversely transcribed into cDNA using PrimeScript™ RT reagent Kit (Takara Biomedical Technology Corporation, Beijing, China). RT‐qPCR was performed on CFX Opus (Bio‐Rad Laboratories Corporation, Shanghai, China) using Talent qPCR PreMix (SYBR Green) (Tiangen Biochemical Technology Corporation, Beijing, China). The complete reactions were subjected to the following program of thermal cycling: 40 cycles of 5 s at 95°C and 15 s at 60°C. Housekeeping gene Gapdh was used for the normalisation of data before the calculation was performed with the 2^−ΔΔCt^ method. Primer sequences (forward and reverse, respectively) were exhibited as follows: CD14(GCGTCGACGCCACCATGAGCCGGCAGGTGGT; GCGGATCCCTACTTGGCCTGAACAGTCTCCT), SPP1(ATCTCACCATTCGGATGAGTCT; ATCTCACCATTCGGATGAGTCT), PRDX5(CCAATCAAGACACACCTGCC; TCTTGAGACGTCGATTCCCA) and GPNMB(GAAATTCATCCGACGAAAC; ATTGGTGGAAACAAACAGG).

### 2.14 Statistical analysis

For bioinformatics analysis, the statistical analysis was conducted using R software (version 4.3.1). Wilcoxon rank‐sum tests were employed to compare values between the test and control groups. Differences among multiple groups were assessed using Kruskal–Wallis's test. To evaluate the diagnostic efficacy of the relevant indicators for SAH, the receiver operating characteristic (ROC) curve and area under the curve (AUC) were employed, with the selection of the threshold based on the highest Youden index to maximize sensitivity and specificity (Youden index = sensitivity + specificity – 1). For PCR data, the data are expressed as the mean ± standard deviation(SD). The GraphPad Prism 9.0 software (GraphPad, San Diego, CA, USA) was used for statistical analysis. The Shapiro–Wilk test was used to test the normality of the distribution of the test data set. Data groups with a normal distribution (two groups) were compared using a two‐sided unpaired Student's *t*‐test. A significance level of *p* < 0.05 was considered statistically significant.

## 3 RESULTS

### 3.1 Single‐Cell transcriptional profiling of macrophages

**FIGURE 2:**
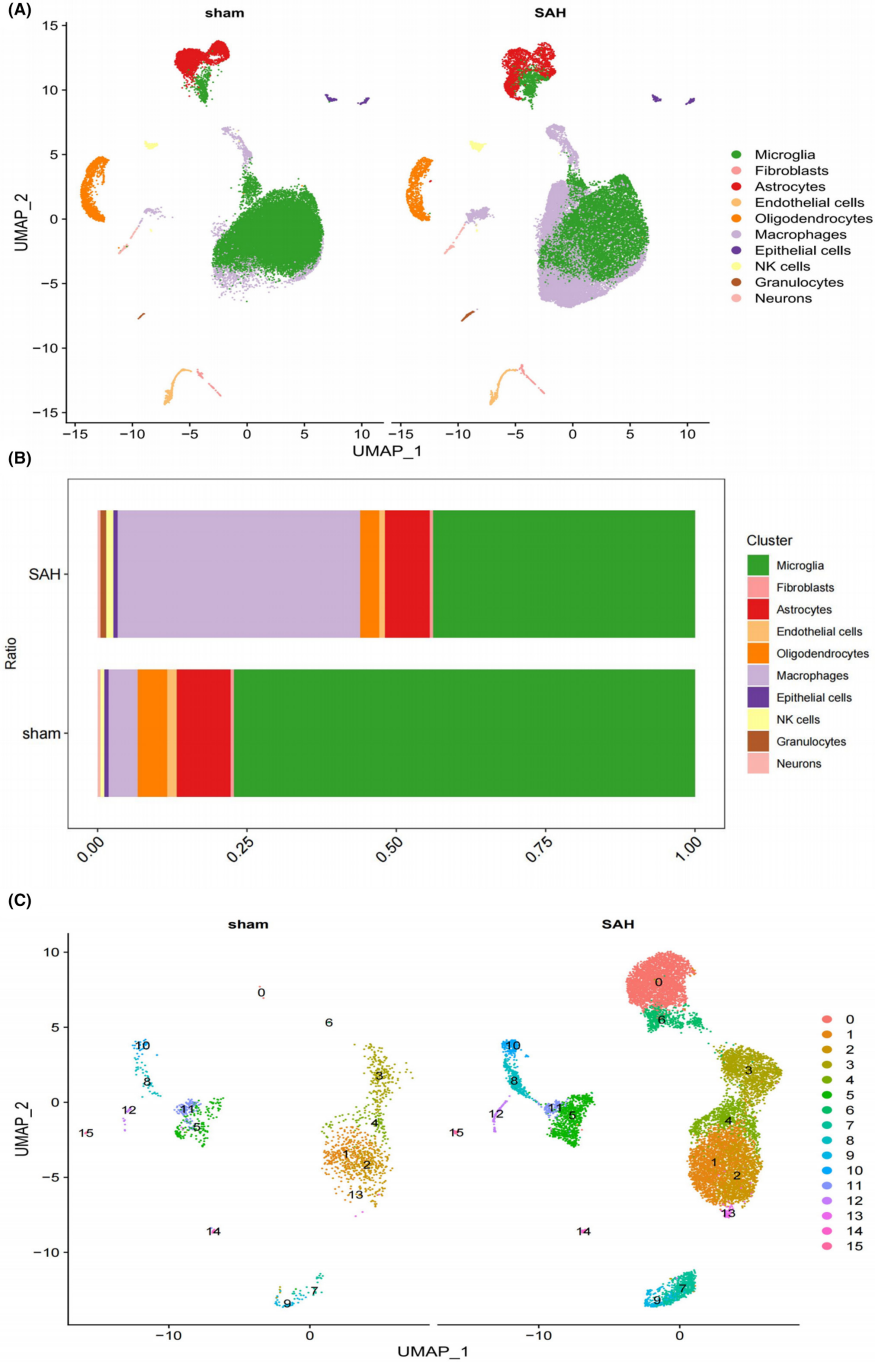
Bioinformatics analysis of single cell transcriptome. (A) The uniform manifold approximation and projection (UMAP) of cell types from SAH and sham samples. (B) A bar chart showing the proportion of various cell types from SAH and sham samples. (C) The uniform manifold approximation and projection (UMAP) of subclusters of Macrophages from SAH and sham samples.

**FIGURE 3:**
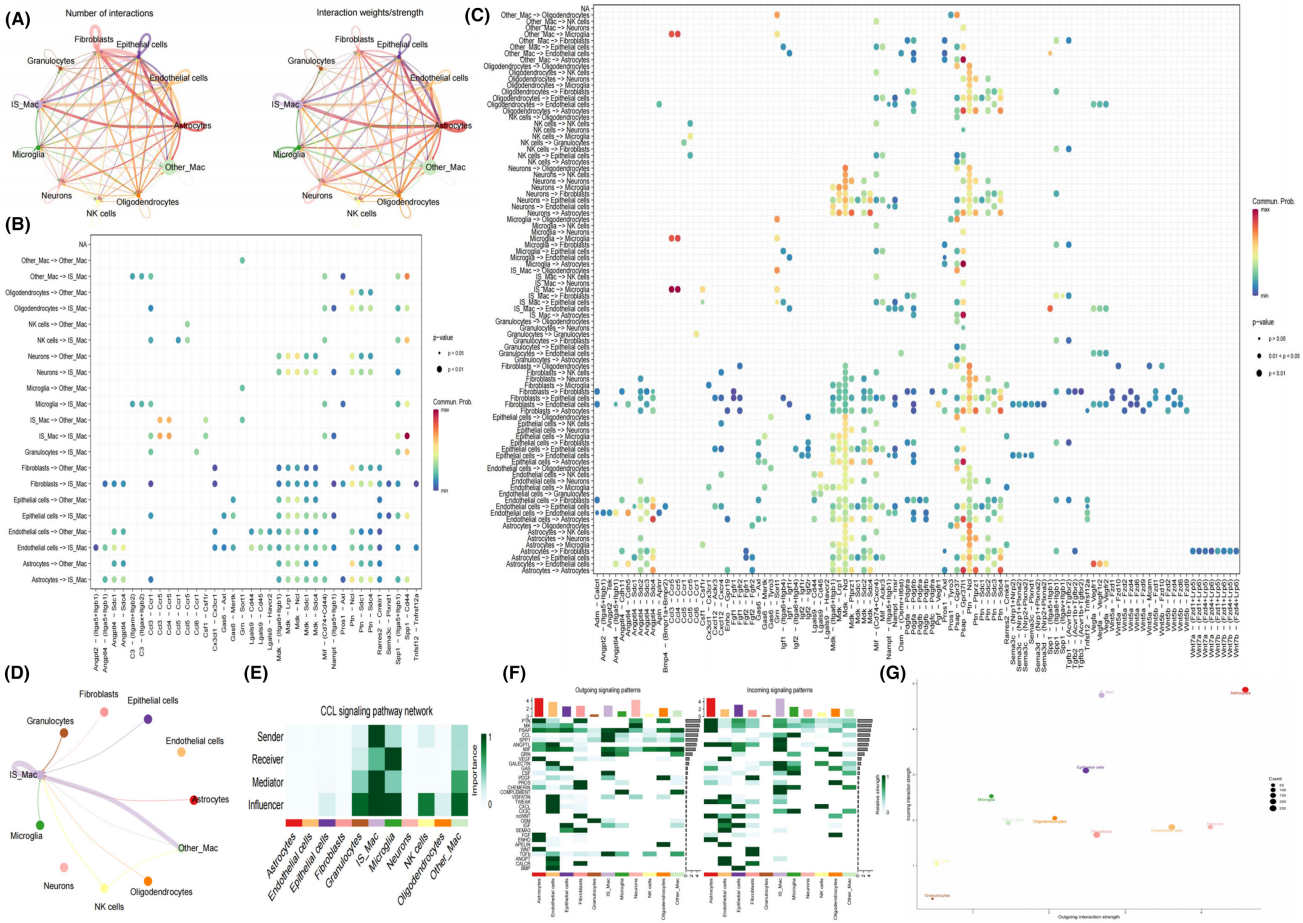
Strength and pathways involved in cell–cell interactions from SAH samples. (A) Interaction net count plot of right temporal cortex cells from SAH samples. The interaction weight plot of each cells. The thicker the line represented, the more the number of interactions, and the stronger the interaction weights/strength between the two cell types. (B, C) Summary of selected ligand–receptor interactions between different cell clusters from SAH samples, respectively. *p‐*Values (permutation test) are represented by the size of each circle. The colour gradient indicates the level of interaction. (D) The analysis of intercellular communication networks using CellChat revealed the inferred interactions involved in CCL signalling pathways. The circle plot visualizes the intercellular communication network for these pathways, highlighting the ligand‐receptor pairs and their connections between different cell populations. (E) This analysis quantifies the relative importance of cell groups in CCL signalling networks using network centrality measures. Influencer cells regulate information flow, while gatekeeper cells control communication between cell groups. Importance is based on sender, receiver, mediator and influencer roles. Darker colours indicate greater involvement in these roles. (F) Heatmaps of different signals contributing mostly to outgoing or incoming signalling of each cell population. (G) The incoming and outgoing strength of each cell population under SAH samples.

We generated single‐cell RNA‐seq profiles for three SAH and three sham groups. After initial quality control, 63,357 single‐cell transcriptomes were obtained, and filtering resulted in the retention of 59,734 cells (Figure S1). To explore cell composition, principal component analysis was performed on 2500 variable genes selected from all cells, revealing 10 major cell clusters, including microglia, fibroblasts, astrocytes, endothelial cells, oligodendrocytes, macrophages, epithelial cells, NK cells, granulocytes and neurons (Figures 2A and 1B). Bar graphs depicting the proportions of each cell type in the SAH and sham groups were generated. Macrophages were selected for further analysis, identifying 15 main clusters based on PCA of the 2500 variable genes from all cells. Cluster 0 and cluster 6 were defined as SAH‐specific macrophages (SSM), while the remaining clusters were categorised as non‐SSM. CellChat analysis revealed diverse interactions among these cell types (Figure 2C). Additionally, interactions between the other nine cell clusters and SSM were significantly more abundant than those between non‐SSM and other cell types (Figure 3A–C). Consequently, we hypothesised that SSM plays a crucial role in SAH disease through paracrine regulation by multiple cell types. The CCL signalling network and related ligand‐receptor interactions exhibited the highest communication probability among SSM interactions (Figure 3D–F). Furthermore, CellChat results demonstrated that SSM exhibited higher outward and inward interaction strengths in SAH, while non‐SSM exhibited lower strengths (Figure 3G).

### 3.2 Construction of hdWGCNA Network

**FIGURE 4:**
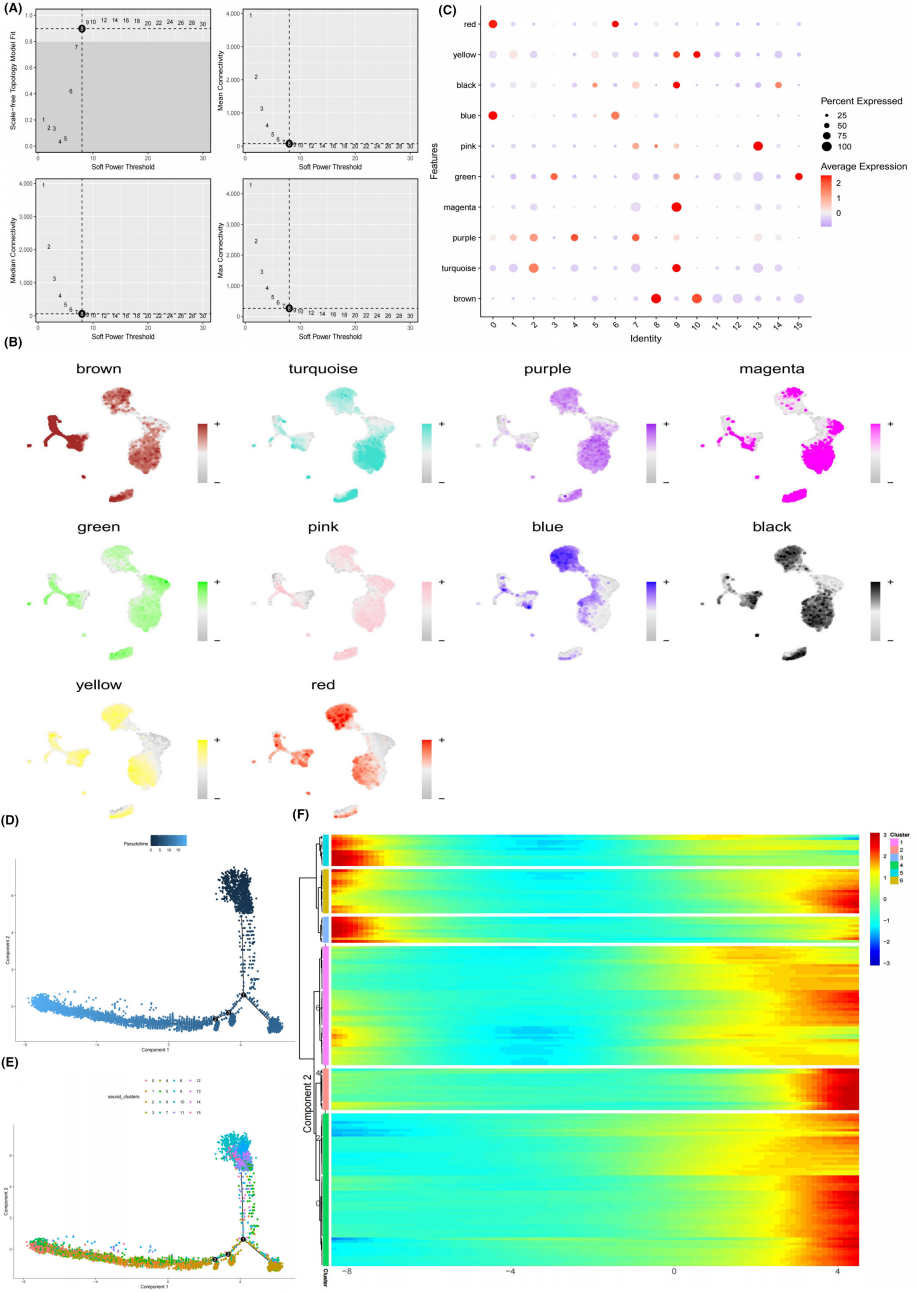
Identification of 160 key genes highly correlated with SAH specific macrophage (SSM). (A) Determination of soft‐threshold power in the WGCNA. (B) Dot plot showing expression of genes in each module in different cluster of macrophage. The size of the dot indicates the percentage of cells within a cell type in which that marker was detected, and its colour indicates the average expression level. (C) Single‐cell sequencing analysis results show the expression in different module eigengenes in macrophages. (D, E) Cell trajectory maps of macrophages highlighting the contribution of cells coming from each state (D) and each cluster. (F) Heatmap showing expression of 160 key genes highly correlated with SSM across single cells. Colour key from blue to red indicates relative expression levels from low to high.

To identify genes highly correlated with SSM, we utilised hdWGCNA to construct co‐expression modules. The power *β* for soft‐thresholding was set to 8, a choice deemed more biologically meaningful and consistent with the scale‐free network (Figure 4A). At *β* = 8, independence was high, and average connectivity was low. Subsequently, a hierarchical clustering tree was generated using *β* = 8, wherein genes with similar expression patterns were grouped into modules via average linkage clustering. A total of 10 modules were identified, distinguished by different colours, and their correlation was illustrated in a heatmap. Notably, the red and blue modules exhibited a high correlation with cluster 0 and cluster 6 (Figures 4B and 3C). Consequently, 80 genes were selected from both the red and blue modules as relevant genes for SSM (Table S1).

Pseudo‐time series analysis indicated that cluster 0 and 6 were in the late stage of development, and the heatmap illustrated that the expression of 160 key genes was predominantly observed in the late stage (Figure 4D–F).

### 3.3 PPI, GO and KEGG pathway enrichment analysis

**FIGURE 5:**
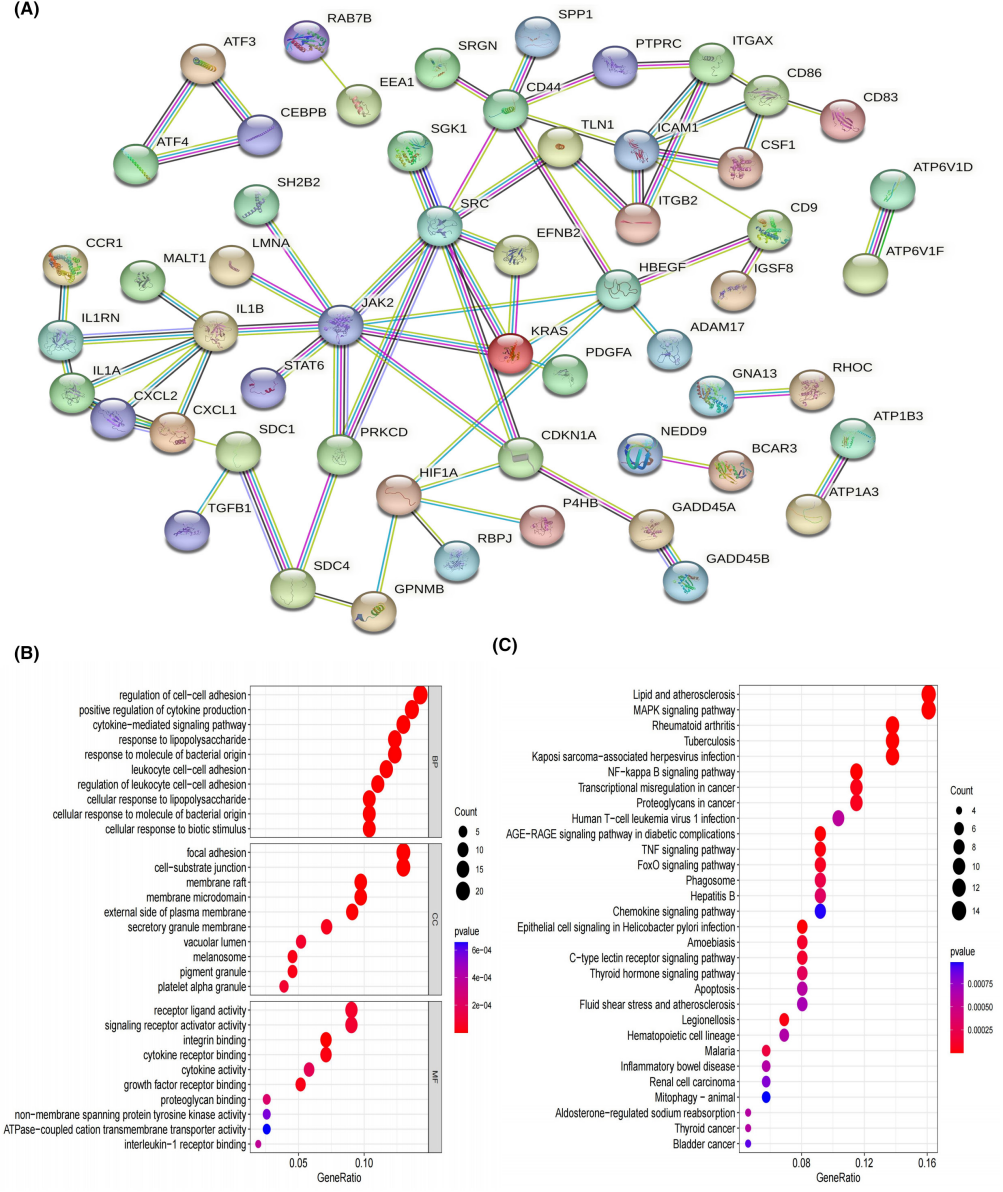
Protein–protein interaction network and Functional analysis. (A) PPI network of 160 key genes highly correlated with SAH specific macrophage (SSM) from hdWGCNA analysis. (B) Bubble graph for Gene Ontology (GO) enrichment. (C) Bubble graph for Kyoto Encyclopedia of Genes and Genomes (KEGG) pathway enrichment (the bigger bubble means the more genes enriched, and the increasing depth of red means the differences were more obvious; *q*‐value: the adjusted *p*‐value).

The PPI network for the identified 160 key genes is presented in Figure 5A. The results of GO analysis revealed that these genes were primarily enriched in processes related to the regulation of cell–cell adhesion, positive regulation of cytokine production, cytokine‐mediated signalling pathway, response to lipopolysaccharide, leukocyte cell–cell adhesion, cell‐substrate junction, focal adhesion, signalling receptor activator activity, and receptor ligand activity (Figure 5B; Table S2).

Moreover, KEGG pathway analysis demonstrated that these genes were predominantly involved in pathways associated with lipid and atherosclerosis, MAPK signalling pathway, NF‐kappa B signalling pathway, TNF signalling pathway and FoxO signalling pathway (Figure 5C; Table S3).

### 3.4 Machine learning

**FIGURE 6:**
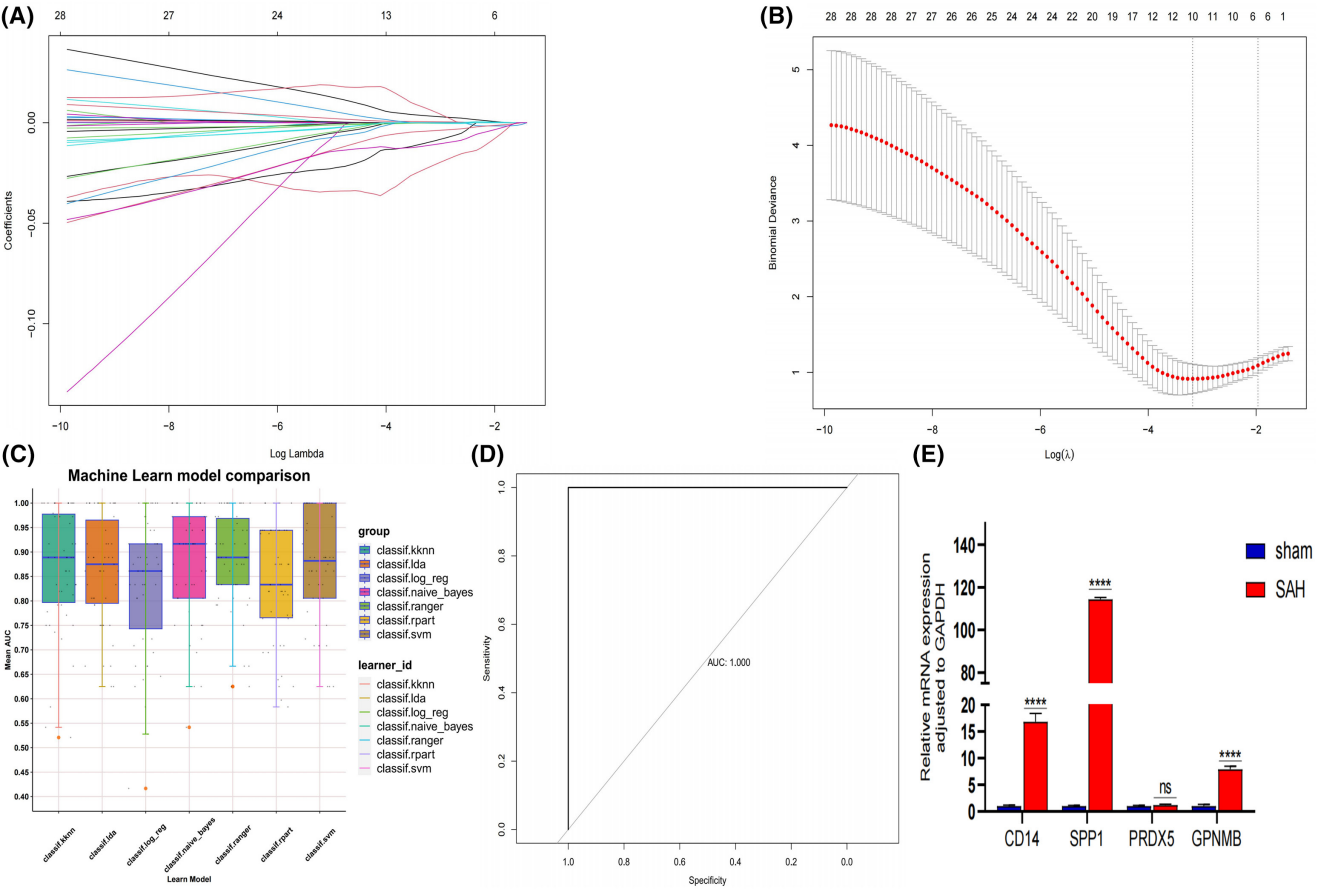
Machine learning for diagnostic model. (A) LASSO coefficient profiles of the 10 SAH‐related genes. (B) A coefficient profile plot was produced against the log (lambda) sequence in the LASSO model. The optimal parameter (lambda) was selected as the first black dotted line indicated. (C) Mean Area Under The Curve (AUC) of the seven machine learning algorithms based on the training set. (D) Receiver operating characteristic (ROC) curve of the random forest algorithm based on the validation set. (E) qPCR showed the gene expression of CD14, SPP1, PRDX5 and GPNMB in the right temporal lobe cortex of the SAH group compared to the sham group. (*p* = 3, ****p < 0.001 vs. sham group, *p* > 0.05 vs. sham group; *t*‐test; mean ± SD).

Applying the hdWGCNA method, we successfully identified 160 key genes highly correlated with SSM. To further refine our selection and choose the most predictive genes, we utilized both univariate logistic regression and LASSO regression methods, ultimately pinpointing 10 genes for in‐depth analysis (Figure 6A,B; Figure S2). Subsequently, employing seven machine learning algorithms, namely logistic regression, linear discriminant analysis, support vector machine, naive Bayes, k‐nearest neighbour, decision tree and random forest, we developed a diagnostic model. The area under the ROC curve (AUC) was calculated for each algorithm (Figure 6C).

Our findings indicated that the random forest algorithm exhibited the most robust predictive performance, boasting an AUC of 0.995 in the training set (GSE36791) and a perfect AUC of 1.000 in the validation set (self‐test data) (Figure 6C,D). These outcomes underscore the high accuracy and reliability of the constructed diagnostic model, suggesting its efficacy in effectively discerning between SAH disease and normal samples.

RT‐pPCR results revealed the increased CD14, SPP1 and GPNMB expression in the SAH rats (Figure 6E).

### 3.5 GSVA Analysis

**FIGURE 7:**
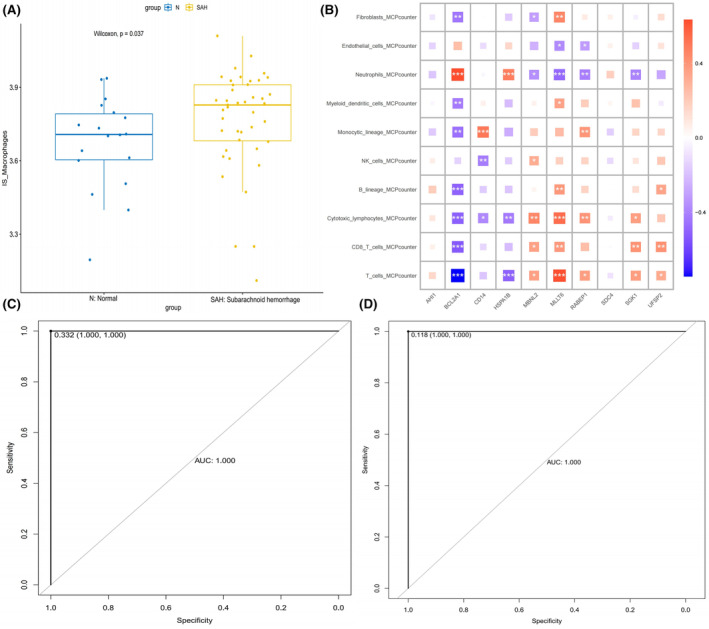
Deep learning for diagnostic model. (A) Bar plots showing distributions of gene set score of 160 key genes related to SAH specific macrophage (SSM) from hdWGCNA analysis in normal and SAH samples. (B) Broad co‐expression network exists between the 10 key genes and the 10 cell types. Red shows a positive correlation and blue shows a negative correlation. *p < 0.05, **p < 0.01, ***p < 0.001. (C) Receiver operating characteristic (ROC) curve of the convolutional neural network algorithm based on the training set. (D) ROC curve of the convolutional neural network algorithm based on the validation set.

The GSVA analysis revealed that SAH samples exhibited higher scores for the SSM subtype compared to the control group, indicative of the prevalence of the SSM subtype in SAH samples (Figure 7A). Subsequently, employing the MCPcounter algorithm, we assessed the presence of various cell types, including T cells, CD8 T cells, cytotoxic lymphocytes, B lineage, NK cells, monocytic lineage, myeloid dendritic cells, neutrophils, endothelial cells and fibroblasts, in the samples based on bulk RNA data. A heatmap was generated to visually represent the correlation between the expression levels of the 10 key genes and these distinct cell types (Figure 7B).

### 3.6 Diagnostic model

Subsequently, we generated an individualized map by incorporating the expression levels of the identified 10 key genes and the composition of 10 distinct cell types in each sample. This individualised map served as the foundation for constructing a diagnostic model employing a 3 × 3 convolutional neural network. Our findings revealed that the model achieved an exceptional AUC of 1.000, with specificity and sensitivity reaching 1 in both the training and validation sets, signifying the model's high accuracy and reliability (Figure 7C,D).

### 3.7 Molecular Docking

Based on the UMAP plot, CD14, CTSK, GPNMB, PRDX5 and SPP1 exhibited robust expression in macrophages of the SAH group, contrasting with minimal expression in macrophages of the sham group. Consequently, we postulated that these genes could serve as potential therapeutic targets for SAH (Figure S3).

**FIGURE 8:**
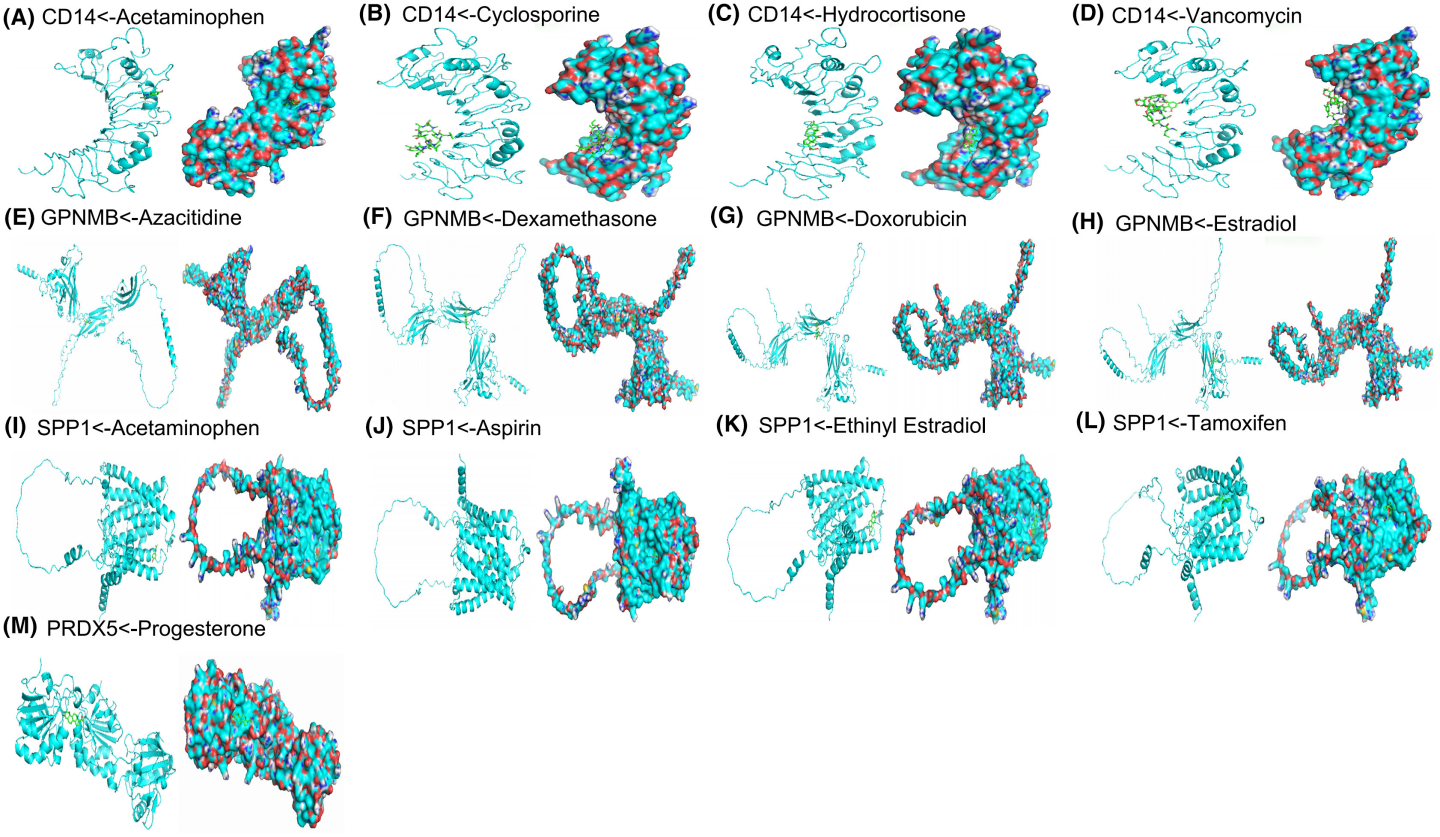
Molecular models of each target gene binding to its predicted drug targets. Acetaminophen target CD14 (A), Cyclosporine target CD14 (B), Hydrocortisone target CD14 (C), Vancomycin target CD14 (D), Azacitidine target GPNMB (E), Dexamethasone target GPNMB (F), Doxorubicin target GPNMB (G), Estradiol target GPNMB (H), Acetaminophen target SPP1 (I), Aspirin target SPP1 (J), Ethinyl Estradiol target SPP1 (K), Tamoxifen target SPP1 (L), Progesteron target PRDX5 (M).

Subsequently, we utilised the CTDbase database to identify chemicals capable of downregulating the expression of these genes in stroke. Noteworthy drugs targeting CD14 comprised Acetaminophen, Cyclosporine, Hydrocortisone and Vancomycin, with affinities of −5.1, −5.4, −7.1 and − 7.1 kcal/mol, respectively. For GPNMB, identified drugs included azacitidine, dexamethasone, doxorubicin and estradiol, with affinities of −6.0, −7.3, −6.9 and − 6.4 kcal/mol, respectively. SPP1‐targeting drugs encompassed acetaminophen, aspirin, ethinyl estradiol and tamoxifen, exhibiting affinities of −5.4, −6.3, −8.7 and − 7.1 kcal/mol, respectively. Additionally, progesterone was identified as a drug targeting PRDX5, with an affinity of −7.1 kcal/mol (Figure 8A–E). Consequently, we propose that these drugs could potentially serve as effective therapeutic interventions for SAH by targeting these specific genes.

## 4 DISCUSSION

Macrophages emerge as pivotal contributors to the intricate pathophysiology of SAH, playing a dual role in both damage and repair processes within the neural microenvironment.^4^, ^5^, ^6^, ^7^, ^23^ These immune cells, residing predominantly in brain tissues and cerebrospinal fluid, orchestrate inflammation, neural damage repair and immune regulation in response to SAH.^24^ While they facilitate the clearance of debris and regulate the inflammatory milieu, overactivation can lead to detrimental effects, perpetuating neural damage through the release of reactive oxygen species and inflammatory mediators.^5^, ^25^, ^26^ The intricate balance in macrophage modulation emerges as a potential target for therapeutic interventions involving anti‐inflammatory drugs, antioxidants and immunomodulatory therapies.

This study employed a rat model of SAH to investigate the right temporal cortex using scRNA‐seq analysis in order to obtain transcriptomic information of different cell types. Specifically, macrophages were selected for subpopulation identification, and SAH‐specific macrophages were discovered and comprehensively characterized. We identified 160 SSM marker genes that exhibited significant expression changes during SAH, which cannot be distinguished using traditional bulk RNA sequencing. Considering the high heterogeneity of diseases, utilising multiple biomarkers rather than a single biomarker enables the establishment of a diagnostic model with higher diagnostic performance. To establish a robust diagnostic model, we combined publicly available database GSE36791 with bulk RNA sequencing data obtained from our self‐constructed rat model of SAH. By applying various machine learning algorithms, we successfully established and validated a diagnostic model consisting of 10 macrophage‐associated molecular features, which accurately diagnosed SAH. The model achieved an AUC of 1.000 in both the training and validation sets, indicating its high diagnostic accuracy. Furthermore, for the first time, we utilized deep learning algorithms, specifically convolutional neural networks, to construct a robust diagnostic model based on the 10 macrophage‐associated molecular features and eight immune cell and two stromal cell features extracted from the samples. The model demonstrated a sensitivity and specificity of 1 in both the training and validation sets, further confirming its superior performance in SAH diagnosis.

The identification of 10 macrophage‐associated molecular features, including AHI1, BCL2A1, CD14, HSPA1B, MBNL2, MLLT6, RABEP1, SDC4, SGK1 and UFSP2, unveils a complex molecular landscape in the context of subarachnoid haemorrhage (SAH). Notably, our UMAP analysis highlighted a significant upregulation of CD14 in macrophages from SAH samples compared to the sham group. This observation aligns with the critical role of CD14 in immune responses and marks it as a potential biomarker and therapeutic target in the SAH milieu.

The subsequent molecular docking results shed light on candidate drugs with the potential to modulate CD14 expression. Hydrocortisone and vancomycin, exhibiting an affinity of −7.1 kcal/mol, emerge as promising candidates for further exploration. Understanding the crucial role of CD14 in immune responses is imperative. As a glycoprotein primarily residing on the cell membrane, its release into body fluids, triggered by bacterial infection or inflammation, initiates inflammatory responses through recognition of pathogen‐associated molecular patterns like LPS.^27^, ^28^, ^29^ CD14's wide expression on macrophages, coupled with its involvement in phagocytosis, clearance of debris and regulation of cytokine production, underscores its hallmark status in macrophage function.^30^ Investigating the intricate interplay between CD14 and macrophages unveils opportunities for leveraging their immunological functions in the context of inflammation and immune‐related disorders.

In the SAH context, where macrophages are activated and accumulate around bleeding sites,^31^, ^32^ CD14's role becomes pivotal. The binding of inflammatory cytokines and pathogen components to CD14 triggers an inflammatory cascade, potentially leading to increased vascular permeability and neuronal damage.^33^, ^34^ Studies indicating an elevated macrophage presence in SAH patients, particularly in the early stages, further emphasise the potential of CD14 as a biomarker for evaluating inflammatory status and disease severity.^35^, ^36^, ^37^ Additionally, CD14 and macrophages contribute to tissue repair processes post‐SAH, highlighting their multifaceted roles in wound healing and neuronal regeneration.^34^

The GPNMB, although not previously studied in SAH, demonstrates significant upregulation in ischemic stroke.^38^, ^39^ Its potential as a therapeutic target, associated with inflammation and neuroinflammation, warrants exploration.^40^ Osteopontin (OPN), or SPP1, plays a dual role in early brain injury and delayed cerebral ischemia in aneurysmal SAH.^41^ Its neuroprotective effects, counterbalanced by potential contributions to chronic hydrocephalus underscore the need for nuanced investigations.^41^ PRDX5, involved in redox balance during brain ischemia–reperfusion, presents a dual role; intracellularly exerting neuroprotective effects and extracellularly inducing pro‐inflammatory responses.^42^

In conclusion, CD14, GPNMB, SPP1, PRDX5 and macrophages stand central in orchestrating inflammatory responses, tissue repair and neuroprotection in SAH. Further exploration of their interaction mechanisms, regulatory roles in macrophage activation and cross‐talk with signalling pathways will deepen our understanding of SAH pathogenesis, paving the way for innovative therapeutic strategies. Targeting these molecules or modulating their expression holds promise for suppressing excessive macrophage activation, mitigating neuroinflammation and ameliorating neuronal damage in SAH.

While our single‐cell analysis provides novel insights, we acknowledge limitations. Sample source constraints, cellular heterogeneity challenges and the need for functional validation underscore the need for comprehensive approaches. Future endeavours, integrating diverse methodologies and addressing technical limitations, will bridge the translational gap, offering tangible clinical implications and therapeutic avenues for SAH.

## 5 CONCLUSIONS

In conclusion, our integration of single‐cell and bulk RNA sequencing has led to the development and validation of a diagnostic model comprising 10 macrophage‐associated molecular features, 8 immune cell types and 2 stromal cell types for SAH patients. The study sheds light on the roles of CD14, GPNMB, SPP1, PRDX5 and macrophages in SAH pathophysiology. These findings offer potential biomarkers and therapeutic avenues for enhancing SAH treatment and prognosis. Further validation and optimisation through basic research and clinical trials are crucial for translating these insights into clinical practice.

## AUTHOR CONTRIBUTIONS

**Sha Yang:** Conceptualization (equal); data curation (equal); formal analysis (lead); methodology (lead); software (equal); validation (equal); visualization (equal); writing – original draft (lead). **Yunjia Hu:** Writing – review and editing (equal). **Xiang Wang:** Data curation (equal); formal analysis (equal); methodology (equal); software (equal); visualization (equal); writing – review and editing (equal). **Mei Deng:** Data curation (equal); software (equal); visualization (equal). **Jun Ma:** Formal analysis (equal); methodology (equal); software (equal). **Yin Hao:** Visualization (equal); writing – review and editing (equal). **Zhongying Ran:** Formal analysis (equal); methodology (equal). **Tao Luo:** Data curation (equal); methodology (equal); software (equal). **Guoqiang Han:** Software (equal); writing – review and editing (equal). **Xin Xiang:** Conceptualization (equal); investigation (equal); project administration (equal); supervision (equal); writing – review and editing (equal). **Jian Liu:** Conceptualization (equal); funding acquisition (equal); investigation (equal); project administration (equal); writing – review and editing (equal). **Hui Shi:** Conceptualization (equal); investigation (equal); project administration (equal); writing – review and editing (equal). **Ying Tan:** Conceptualization (lead); funding acquisition (lead); investigation (equal); project administration (equal); writing – review and editing (equal).

## FUNDING INFORMATION

This work was supported by the National Natural Science Foundation of China (82,360,482, 82,360,376 and 82,260,533), the Guizhou Provincial Science and Technology Projects ([2020]1Z066), Guizhou Provincial People's Hospital Doctor Foundation ([2018]06 and [2018]03), Guizhou Provincial People's Hospital National Science Foundation (GPPH‐NSFC‐2019‐18, GPPH‐NSFC‐2019‐09 and GPPH‐NSFC‐D‐2019‐17), General Project of Chongqing Natural Science Foundation (CSTB2023NSCQ‐MSX0749), Guizhou Provincial People's Hospital Youth Fund (GZSYQN202202).

## CONFLICT OF INTEREST STATEMENT

The authors declare that the research was conducted in the absence of any commercial or financial relationships that could be construed as a potential conflict of interest.

## CONSENT FOR PUBLICATION

Not applicable.

## Supporting information


Figure S1.

Figure S2.

Figure S3.

Table S1.

Table S2.

Table S3.

## Data Availability

Publicly available datasets were analysed in this study. This data can be found here: GEO database (https://www.ncbi.nlm.nih.gov/geo/). Our data can also be found in GEO database.
