# Supplementary figures and images for: Machine learning and deep learning to identifying subarachnoid haemorrhage macrophage‐associated biomarkers by bulk and single‐cell sequencing
Source: J Cell Mol Med. 2024 May 4;28(9):e18296. doi: 10.1111/jcmm.18296 (PMC11069052; doi:10.1111/jcmm.18296)

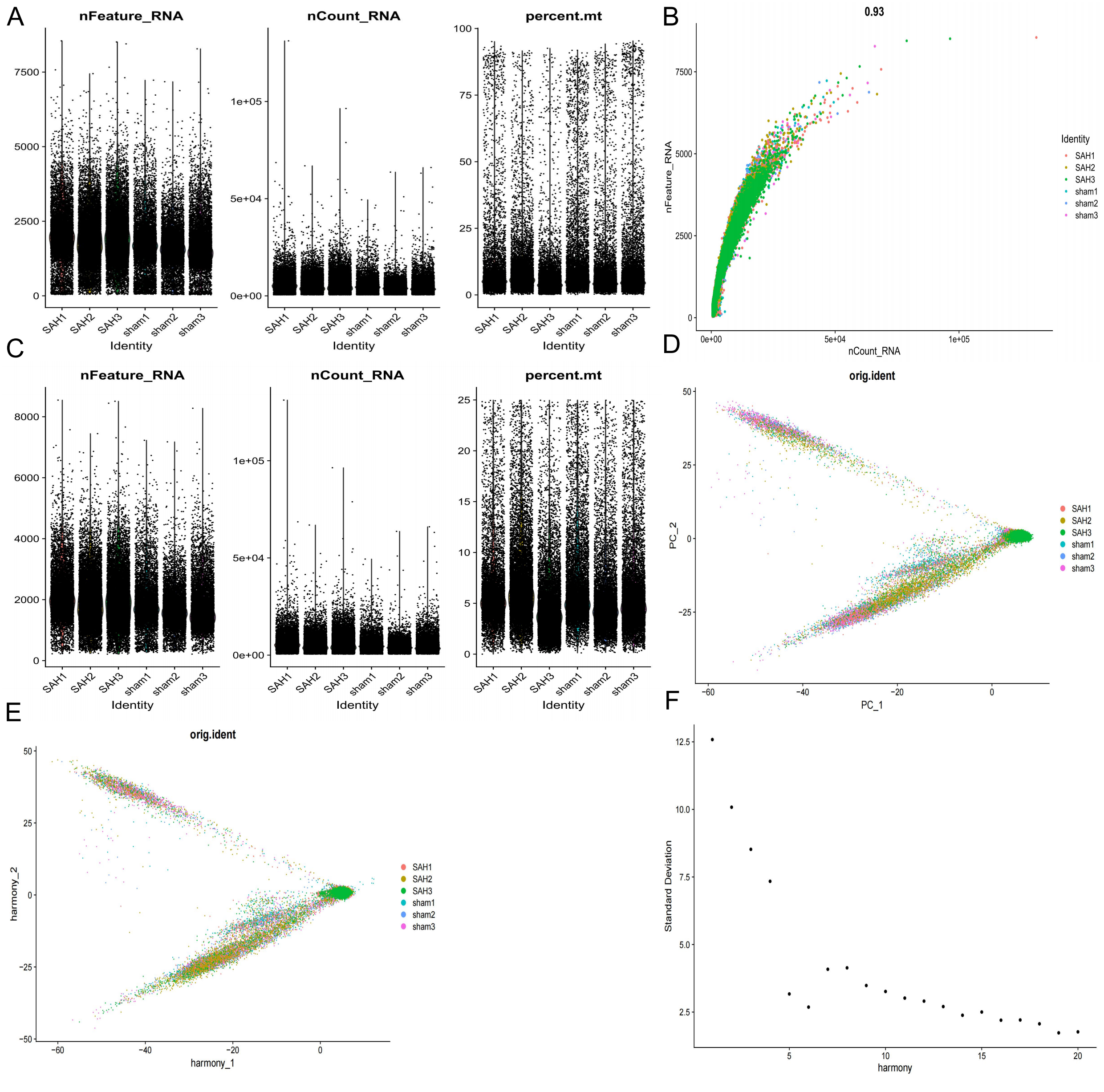

Supplement: Supplementary file 1 — Figure S1. [file JCMM-28-e18296-s003.tif]

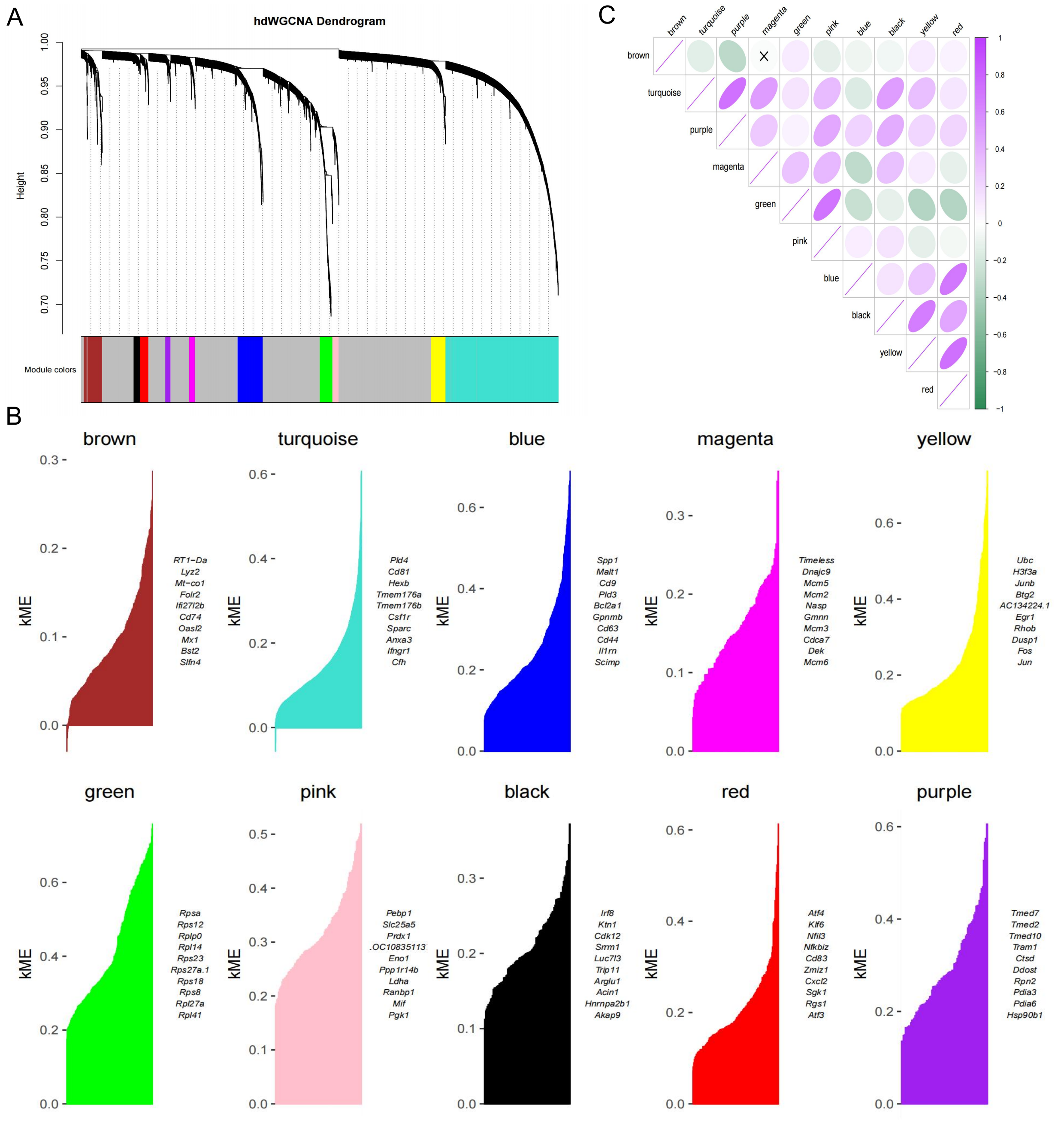

Supplement: Supplementary file 2 — Figure S2. [file JCMM-28-e18296-s001.tif]

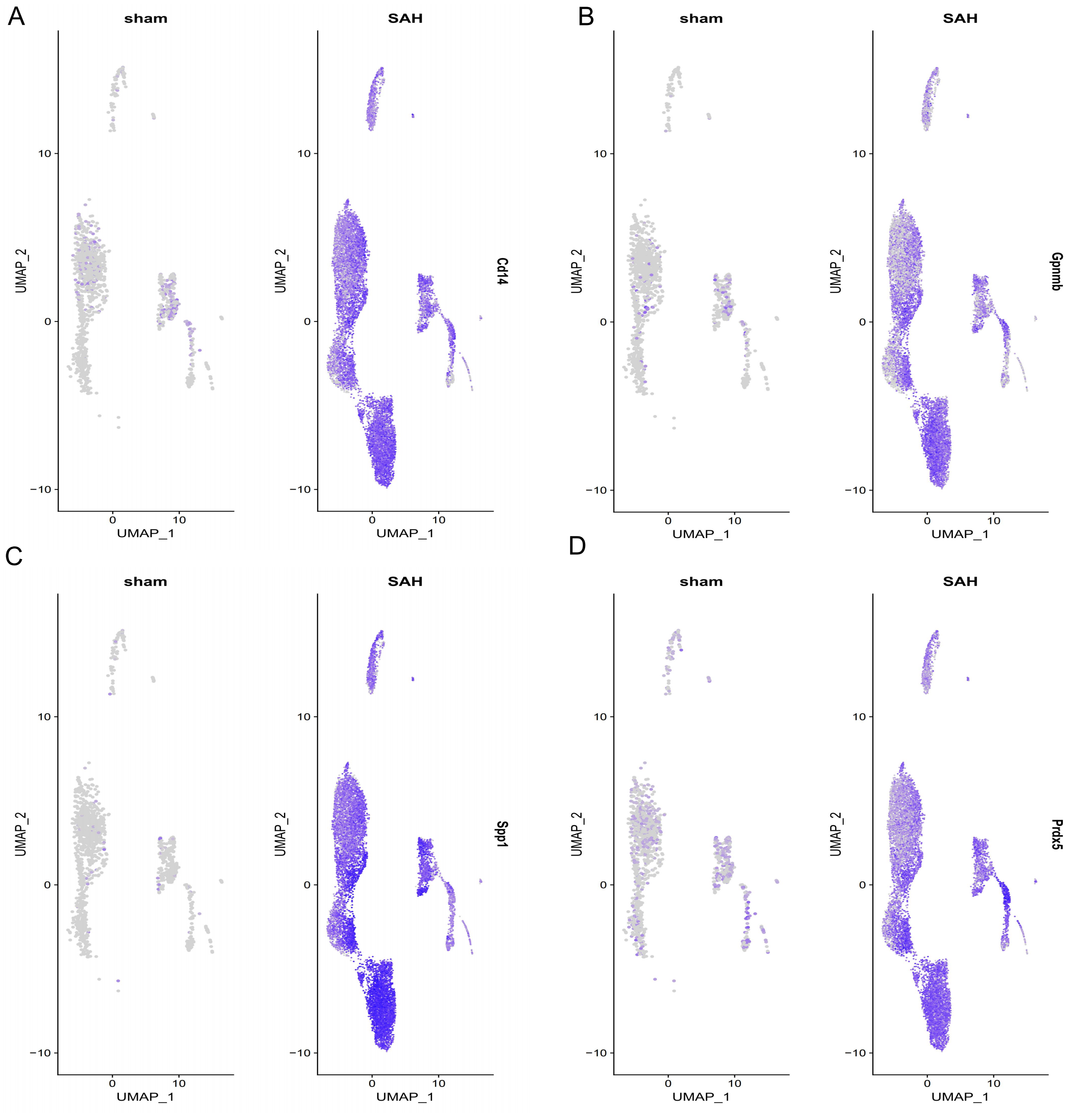

Supplement: Supplementary file 3 — Figure S3. [file JCMM-28-e18296-s006.tif]
